# Sirtuin 1, as a potential prognosis marker in clear cell renal cell carcinoma, regulates lipid metabolism and immune infiltration

**DOI:** 10.17305/bb.2024.10304

**Published:** 2024-08-01

**Authors:** Xuefei Wang, Fangqi Deng, Jiexi Liu, Jiayu Wang, Qing Chen, Jiabin Lu

**Affiliations:** 1 State Key Laboratory of Oncology in South China, Collaborative Innovation Center for Cancer Medicine, Sun Yat-sen University Cancer Center, Guangzhou, China; 2 Department of Pathology, Sun Yat-sen University Cancer Center, Guangzhou, China; 3 Department of Comprehensive Traditional Chinese Medicine, Sun Yat-sen University Cancer Center, Guangzhou, China

**Keywords:** Renal clear cell carcinoma (ccRCC), Sirtuin 1 (Sirt1), prognosis, lipid metabolism disorder, immune infiltration

## Abstract

Clear cell renal cell carcinoma (ccRCC) is a malignancy with a dismal prognosis, caused by the buildup of fat and glycogen. Sirtuin 1 (Sirt1) is a deacetylase that regulates lipid metabolism. In this study, we collected tumor and paracancer tissues from 386 ccRCC patients and followed their prognosis over an extended time period. The expression of Sirt1 in these tissues was assessed using immunohistochemistry, and LinkedOmics database analysis identified differentially expressed genes associated with Sirt1. The survival curve was generated using the Kaplan–Meier method, and immune infiltration was analyzed using the Tumor Immune Estimation Resource (TIMER) web tool. Our findings revealed that Sirt1 was expressed in tumor tissues, but not in normal tissues, and its high expression was associated with a worse prognosis. Furthermore, we observed a positive correlation between high Sirt1 expression and perirenal fat (PF) invasion and necrosis, leading to poorer survival outcomes. We established a nomogram to predict prognosis, and a correlation was observed with immune infiltration. In conclusion, our results suggest that high Sirt1 expression is associated with lipid metabolism disorder and immune infiltration, ultimately contributing to a dismal prognosis in ccRCC.

## Introduction

The incidence of renal cell carcinoma (RCC) has been on the rise, with over 400,000 new cases reported worldwide in 2020, particularly in males [1]. In China, the incidence and mortality rates of RCC were 66,800 and 23,400, respectively, in 2015, with males accounting for almost two-thirds of cases [2]. Despite advances in treatment, RCC remains highly lethal. The majority of primary renal cancers can be classified into three subtypes: clear cell RCC (ccRCC), papillary RCC, and chromophobe RCC. ccRCC and papillary RCC arise from proximal tubule epithelial cells, whereas chromophobe RCC and collecting duct tumors arise from the distal tubules [3]. Moreover, 80% of RCC is ccRCC, which is characterized by lipid and glycogen accumulation. So, lipid metabolism remodeling is significant in ccRCC [4]. The loss of chromosome 3p and von Hippel–Lindau (VHL) function are well-known causes of ccRCC [5]. The deletion of chromosome 3p causes lots of essential genes related to lipid metabolism to be lost or haploinsuffcient. And VHL protein is a main ubiquitase ligand for ubiquitinylating and degradation of hypoxia-induced factors (HIFs) under hypoxia conditions. The reduction of VHL and increase of HIFs cause lipid droplets (LDs) accumulation in ccRCC [4, 5]. Additionally, perirenal fat (PF) surrounds the kidney and plays a crucial role in kidney function. According to reports, PF invasion (PFI) occurs in 26% of cases of ccRCC and has a poor prognosis [6]. Wei et al. report that bidirectional communication between ccRCC tumor cells and perinephric fat promotes the growth, invasion, and metastasis of the former [7]. Despite the importance of the connection between ccRCC and lipid metabolism, this area of research has not received sufficient attention. Therefore, investigating novel pathways of lipid metabolism modification in ccRCC is essential to prevent the oncogenesis and progression of this disease.

Sirtuins, a family of nicotinamide adenine dinucleotide (NAD)-dependent protein deacetylases, have been extensively studied due to their catalytic activities [8]. And Sirt1, 2, 3, 4, 5, and 6 are members of this family and have these catalytic activities [9]. Among them, Sirtuin 1 (Sirt1) is a highly conserved mammalian homolog of Sir2 [10] and has been the subject of numerous research studies. The link between Sirt1 and energy metabolism has been extensively investigated, particularly in relation to fat accumulation and gluconeogenesis. For example, Sirt1 represses Peroxisome proliferator-activated receptor gamma (PPARG) in adipocytes and influences fat accumulation [11, 12]. And in the liver, it is discovered that Sirt1 modulates different pathways to modify gluconeogenesis [13]. Recently, there have been many studies that focus on the relationship between Sirt1 and inflammation, especially on nuclear factor kappa B (NF-κB). The NF-κB is a family of transcription factors and is considered a major regulator of the inflammatory responses due to its ability to regulate the transcription of genes involved in the establishment of immune and inflammatory response in many types of cells [14]. On the one hand, Sirt1 has been shown to acetylate and suppress NF-κB, thereby repressing inflammation in the liver [15]. On the other hand, Sirt1 protein levels are downregulated by IL1β/NF-κB signaling in acetaminophen (APAP) hepatotoxicity, resulting in inflammation and oxidative stress [16]. Additionally, Sirt1 has been implicated in various types of cancer, including lung cancer, breast cancer, gastric cancer, colon cancer, liver cancer, pancreatic cancer, ovarian carcinoma, cervical cancers, prostate cancer, lymphoma, and leukemia, carcinoma of the head and neck, brain glioma, soft tissue sarcomas, and skin cancer [17]. However, the role of Sirt1 in RCC remains poorly understood. Given that ccRCC is a cancer with abnormal lipid accumulation and Sirt1 is considered an influencing factor of metabolism, this article aims to investigate the effect of Sirt1 in ccRCC and provide new ideas and methods for the diagnosis and treatment of ccRCC.

## Materials and methods

### Patients and clinical materials

This study included a total of 386 patients diagnosed with ccRCC who underwent surgical resection at Sun Yat-sen University Cancer Center between 2010 and 2015. The diagnosis of ccRCC was confirmed by a pathological examination of the surgical specimen’s clinical data, including age, sex, and tumor stage, which were collected from medical records. The tumor stage was determined according to the International Society of Urological Pathology’s international consensus conference in 2012 [18].

### Immunohistochemistry (IHC)

The paraffin-embedded tissue samples are made into chips with tumor and paracancer tissues. These chips were cut into 4-µm-thick sections and mounted on poly-L-lysine-coated slides. The sections were deparaffinized in xylene and rehydrated in a graded series of ethanol solutions. Antigen retrieval was performed by heating the sections in 10 mM EDTA buffer (pH 9.0) in a 100 ^∘^C pressure cooker for 3 min. Endogenous peroxidase activity was blocked by incubating the sections in 3% hydrogen peroxide for 10 min. The sections were then incubated with primary antibodies against Sirt1 (1:100 dilution, OriGene, US) at 37 ^∘^C for 50 min. After washing with phosphate-buffered saline (PBS), the sections were incubated with horseradish peroxidase-conjugated secondary antibodies (DAKO, Denmark) for 30 min at room temperature. The sections were then stained with 3,3’-diaminobenzidine (DAB) and counterstained with hematoxylin. The stained sections were observed under a light microscope and images were captured using a digital camera. Staining intensity was scored 0 (negative), 1 (weak), 2 (moderate), and 3 (strong). Staining range was scored on a 4-point scale (0 ═ 0%, 1 ═ 1%∼24%, 2 ═ 25%∼49%, 3 ═ 50%∼74%, and 4 ═ 75%∼100%). The final staining score is the staining intensity score × the staining range score.

### Bioinformatics analyses

We utilized the “rms” package in R to develop nomograms. Receiver operating characteristic curve (ROC) analysis was carried out using the “pROC” package and “timeROC” package, and the analysis results were visualized with the “ggplot2” package. Protein–protein interaction networks functional enrichment analysis was built using the Search Tool (STRING) (version 11.5) (https://string-db.org/) to search for interacting genes [19].

### Web analytics

Tumor Immune Estimation Resource (TIMER, cistrome.shinyapps.io/timer) allows users to explore the relationship between the expression of certain genes and the degree of immune infiltration in various cancer types [20]. It utilizes data from The Cancer Genome Atlas (TCGA) database. By using the TIMER database, we analyzed the relationship between Sirt1 expression and immune infiltration.

The LinkedOmics portal provides access to multi-omics data from all 32 TCGA cancer types [21]. Through this website, RNA-seq datasets for clear cell RCC (ccRCC) from TCGA can be easily accessed. We chose all 533 RNA high-throughput sequencing samples from the TCGA database that had a ccRCC histological categorization. Based on the determination of Sirt1’s Spearman correlation coefficients with other genes, the Kyoto Encyclopedia of Genes and Genomes (KEGG) and Gene Set Enrichment Analysis (GSEA) identified significant associations between gene sets and specific biological pathways.

### Ethical statement

This study was approved by the Ethics Committee of Sun Yat-sen University Cancer Center, China. The number of Ethics Approval is B2023-117-01. The informed consent was obtained from all subjects and/or their legal guardian(s). All methods were carried out in accordance with relevant guidelines and regulations.

### Statistical analysis

The majority of statistical analysis was calculated by GraphPad Prism. Data are presented as the mean ± standard error. Unpaired two-tailed *t*-tests yielding a *P* value <0.05 indicated a statistically significant difference. Overall survival (OS) analysis and progression-free survival (PFS) analysis were performed by Kaplan–Meier plots and the differences were compared using the log-rank test. The best cut-points were analyzed by X-tile [22].

## Results

### Sirt1 expression in ccRCC

**Figure 1.**
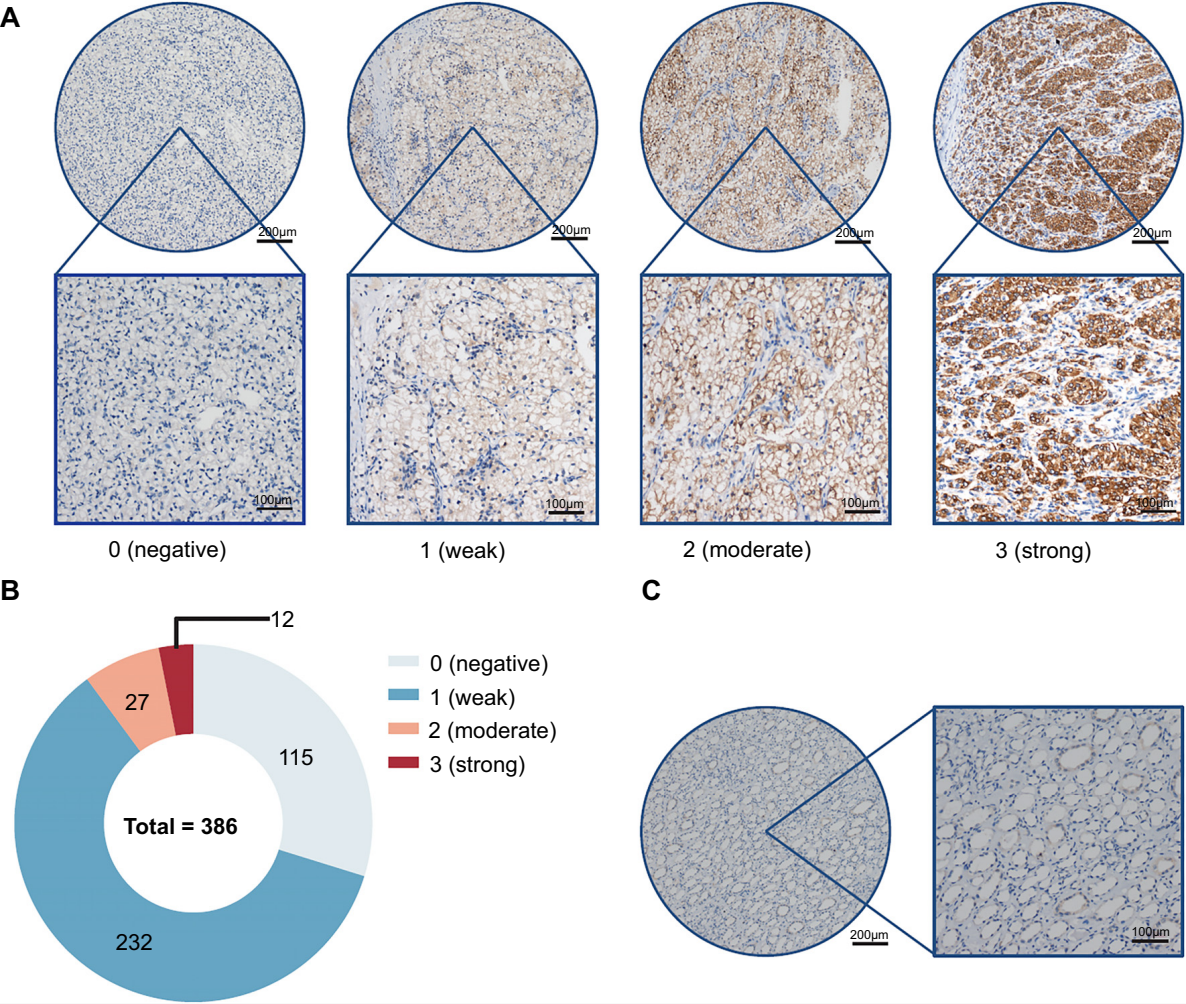
**Sirt1 expression in ccRCC.** (A) Representative immunohistochemistry images of Sirt1 in different expression intensities of ccRCC cancer tissues; (B) The proportion of Sirt1 different staining intensities; (C) Immunohistochemistry images of Sirt1 in normal tissues. Sirt1: Sirtuin 1; ccRCC: Clear cell renal cell carcinoma.

We first detected the expression of Sirt1 in ccRCC. Aimed at this purpose, we collected the paraffin blocks from 386 patients suffering from ccRCC. Two cancer tissue and paracancer tissue paraffin blocks were selected from each patient according to microscopical morphology. These paraffin blocks were made into chips and IHC stained. Two professional pathologists read these Sirt1 IHC stain sheets. To standardize IHC staining results, the staining strength was evaluated as negative (scored 0), weak (scored 1), moderate (scored 2), and strong (scored 3) (Figure 1A). The staining range was evaluated as 0% (scored 0), 1%∼24% (scored 1), 25%∼49% (scored 2), 50%∼74% (scored 3), and 75%∼100% (scored 4). Multiplying the numbers in parentheses between the two evaluated items gets the final evaluated score (0–12). The results of the two pathologists were averaged for statistical purposes. In cancer tissue, Sirt1 was expressed in 79% (Figure 1B) whereas it was scarcely present in nearly all normal tissue (Figure 1C).

**Figure 2.**
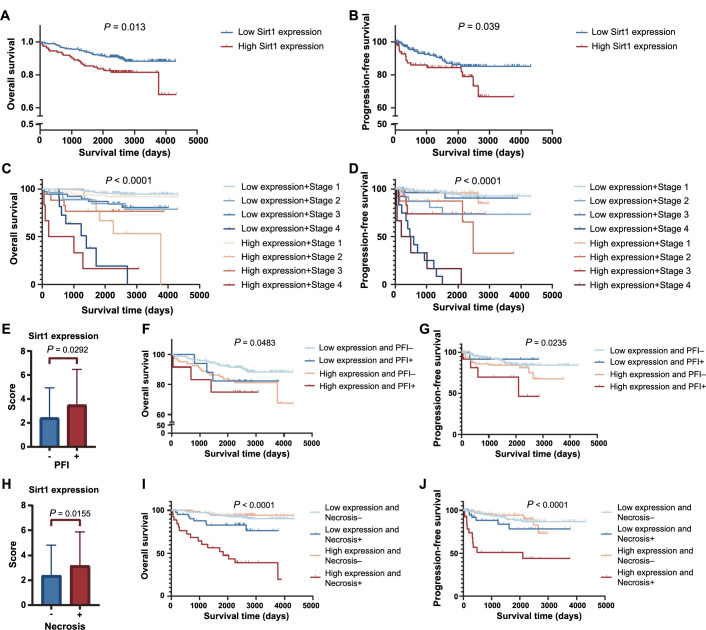
**Poor prognosis for patients with high Sirt1 expression**. (A) The different levels of Sirt1 expression were analyzed using Kaplan–Meier survival analysis to determine OS; (B) PFS in relation to varying levels of Sirt1 expression was determined by Kaplan–Meier survival analysis. Kaplan–Meier survival analysis shows OS (C) and PFS (D) in different Sirt1 expression groups and different stages; (E) Sirt1 expression is higher in PFI patients; (F) Kaplan–Meier survival analysis reveals OS in different Sirt1 expression groups and with or without PFI; (G) Kaplan–Meier survival analysis reveals OS in different Sirt1 expression groups and with or without PFI; (H) Sirt1 expression is higher in necrosis patients; (I) Kaplan–Meier survival analysis reveals OS in different Sirt1 expression groups and with or without necrosis; (J) Kaplan–Meier survival analysis reveals OS in different Sirt1 expression groups and with or without necrosis. *P* value <0.05 indicates a statistically significant difference. Sirt1: Sirtuin 1; OS: Overall survival; PFS: Progression-free survival; PFI: Perirenal fat invasion.

High expression of Sirt1 is associated with a poor prognosis. We present experimental evidence on the survival outcomes of patients with varying Sirt1 expression levels. Based on the latest follow-up data in 2022, we conducted a survival analysis and found that using the median score as a cut-off to divide patients into two groups did not yield statistically significant results. To determine the most appropriate cut-off point, we utilized X-tile software for analysis. The software divided patients into two groups based on a score of <═3.5 (low expression, *n* ═ 288) and >3.5 (high expression, *n* ═ 98). Applying this grouping, patients exhibiting a tumor expressing high levels of Sirt1 had lower OS (Figure 2A) and lower PFS (Figure 2B).

Upon further analysis, we observed no significant difference in Sirt1 expression levels among patients in different stages. However, we did observe significant differences in OS and PFS among patients with varying Sirt1 expression levels and stages. Specifically, patients in stage 1 (low expression, *n* ═ 201; high expression, *n* ═ 64) ccRCC with low mortality did not exhibit any significant influence of Sirt1 expression on their survival outcomes. In contrast, patients in stages 2 (low expression, *n* ═ 28; high expression, *n* ═ 9) and 3 (low expression, *n* ═ 39; high expression, *n* ═ 19) with higher Sirt1 expression levels had significantly poorer prognoses than those with lower expression levels. Due to the limited number of cases and high mortality rates in severe cases, it was difficult to obtain statistically significant results for stage 4 (low expression, *n* ═ 11; high expression, *n* ═ 6) patients (Figure 2C). The trend observed in the PFS results was similar to that of the OS results across different stages (Figure 2D). Overall, our findings suggest that Sirt1 expression levels only affect the survival outcomes of patients in the middle stages.

### Other factors influencing prognosis and construction of clinical prognostic model

PFI serves as a crucial prognostic indicator in the TMN stage (AJCC, 8th edition). Its association with a poorer prognosis has been well-established [23]. And our study shows the same results in the OS and PFS. Sirt1 functions as a deacetylase and regulates fat metabolism [8]. To elucidate the relationship between PFI and Sirt1 expression, we conducted a comparative analysis of Sirt1 expression levels in patients with and without PFI. Our findings indicate that patients with PFI exhibit higher levels of Sirt1 expression (Figure 2E). Furthermore, combined with OS and PFS in different patients, high expressing Sirt1 is related to PFI occurrence and with a worse prognosis (Figure 2F and 2G) (Low expression and PFI −, *n* ═ 253; Low expression and PFI +, *n* ═ 17; High expression and PFI −, *n* ═ 82; High expression and PFI +, *n* ═ 13).

Tumor necrosis is an independent poor prognostic indicator [24] and a higher necrosis rate is associated with a significantly higher risk of recurrence [25]. According to the study, the regulation of Sirt1 levels and its activation play a crucial role in mediating the multistep process of drug-induced liver injury (DILI), which contributes to the development of DILI, and subsequently triggers severe oxidative stress, inflammation, and apoptosis. These processes collectively lead to hepatocellular necrosis and ultimately result in liver damage [26]. Nevertheless, the impact of Sirt1 on tumor necrosis in ccRCC remains unexplored. Our observations have revealed a significant association between Sirt1 expression and necrosis (Figure 2H). And both lead to worse prognosis (Figure 2I and 2J) (Low expression and Necrosis −, *n* ═ 244; Low expression and Necrosis +, *n* ═ 43; High expression and Necrosis − *n* ═ 73; High expression and Necrosis +, *n* ═ 36).

**Table 1:** The relevant parameters for each variable in the nomogram of overall survival

	**Coef**	**S.E.**	**Wald Z**	**Pr (>|Z|)**	**HR**
Age	−0.0352	0.0131	−2.69	0.0071	1.0357025
Gender ═ male	−0.5141	0.3460	−1.49	0.1373	1.9361806
Stage	−0.9151	0.1617	−5.66	<0.0001	2.5292246
Necrosis ═ yes	−1.1250	0.3502	3.21	0.0013	2.5863738
PFI ═ yes	0.9438	0.4956	1.90	0.0569	0.3641643
Sirt1 ═ High_exp	−0.2483	0.3166	−0.78	0.4330	1.6491216
Nuclear_grade	−0.7329	0.2845	−2.58	0.0100	1.9708272

Coef: Coefficients; S.E.: Standard error; Wald Z: Wald test critical value; Pr: Probability; HR: Hazard ratio; PFI: Perirenal fat invasion.

**Table 2:** The relevant parameters for each variable in the nomogram of progression-free survival

	**Coef**	**S.E.**	**Wald Z**	**Pr (>|Z|)**	**HR**
Age	−0.0463	0.0162	−2.87	0.0042	1.0455270
Gender ═ male	0.1069	0.3578	0.30	0.7652	0.8069903
Stage	−1.0540	0.1760	−5.99	<0.0001	3.0162504
Necrosis ═ yes	−0.5208	0.3960	−1.32	0.1885	1.3143747
PFI ═ yes	1.1523	0.5910	1.95	0.0512	0.3411541
Sirt1_expression_score	−0.1233	0.0651	−1.89	0.0584	1.1568529
Nuclear_grade	−1.0183	0.3264	−3.12	0.0018	3.0878866

Coef: Coefficients; S.E.: Standard error; Wald Z: Wald test critical value; Pr: Probability; HR: Hazard ratio; PFI: Perirenal fat invasion.

**Figure 3.**
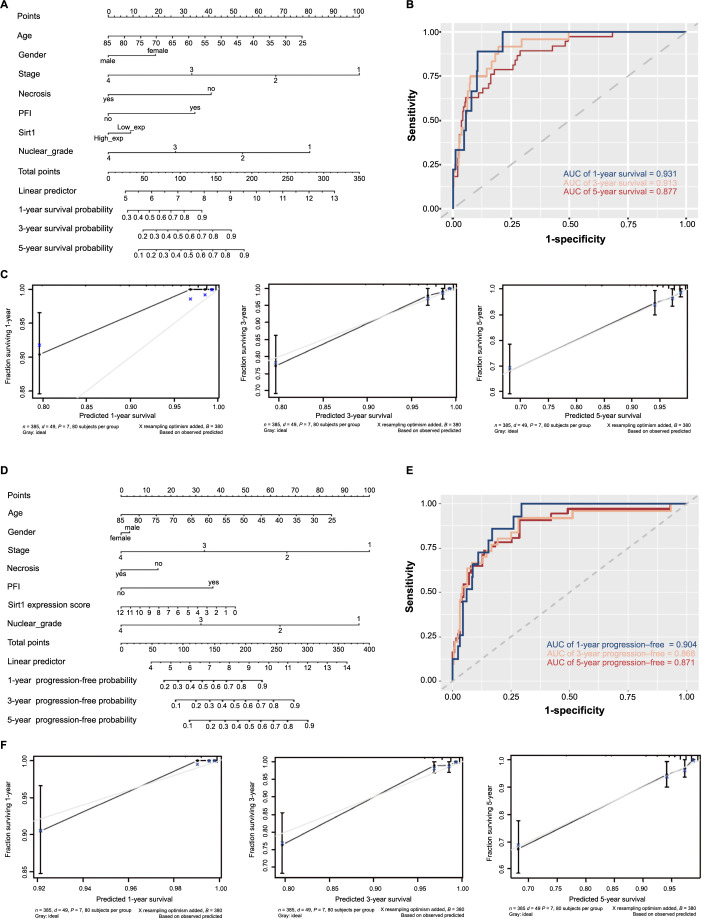
**Predicting the prognosis of ccRCC patients**. (A) Nomogram prediction model based on the OS data of ccRCC patients; (B) ROC analysis based on nomogram prediction model of OS; (C) Calibration analysis in different time frames based on nomogram prediction model of OS; (D) Nomogram prediction model based on the PFS data of ccRCC patients; (E) ROC analysis based on nomogram prediction model of PFS; (F) Calibration analysis in different time frames based on nomogram prediction model of PFS. OS: Overall survival; PFS: Progression-free survival; ROC: Receiver operating characteristics; ccRCC: Clear cell renal cell carcinoma; AUC: Area under the curve.

We subsequently devised a prognostic nomogram based on the outcomes of a multivariate Cox analysis of patients’ OS data (Figure 3A). The time-dependent ROC curve area for prognoses at the 1-year, 3-year, and 5-year marks were 0.931, 0.913, and 0.877, respectively (Figure 3B). Moreover, the reliability of this model in 3-year and 5-year periods was confirmed by calibration analysis (Figure 3C). Using the same methodology, we constructed a nomogram for PFS (as shown in Figure 3D). The time-dependent ROC curve area for prognoses at the 1-year, 3-year, and 5-year marks were 0.904, 0.868, and 0.871, respectively (Figure 3E). Calibration analysis further confirmed the reliability of this model for prognoses at 1-year, 3-year, and 5-year marks (Figure 3F). The coefficients, standard deviations, Wald values, *P*-values, and risk ratios for each variable are shown in Tables 1 and 2. These clinical models can help doctors and researchers better assess patients’ health risks, and improve the accuracy and efficiency of diagnosis, resulting in better healthcare for patients.

### Potential mechanisms associated with Sirt1 affecting the prognosis

**Figure 4.**
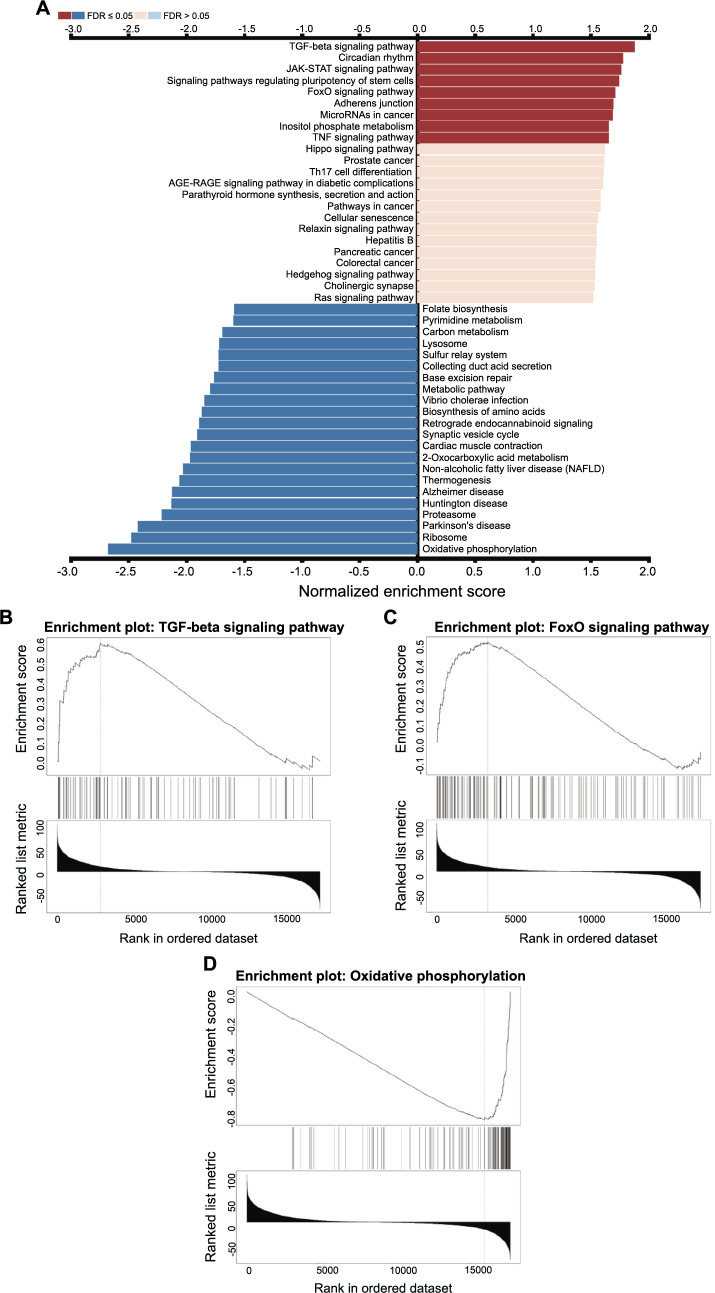
**The GSEA results**. (A) To identify significant associations between Sirt1 and specific biological pathways, 533 RNA high-throughput sequencing samples from the TCGA database that had a renal clear cell carcinoma histological categorization was analyzed by the KEGG and GSEA at LinkedOmics website; (B–D) High enrichment score gene sets in GSEA. GSEA: Gene set enrichment analysis; TCGA: The Cancer Genome Atlas; KEGG: Kyoto Encyclopedia of Genes and Genome.

**Figure 5.**
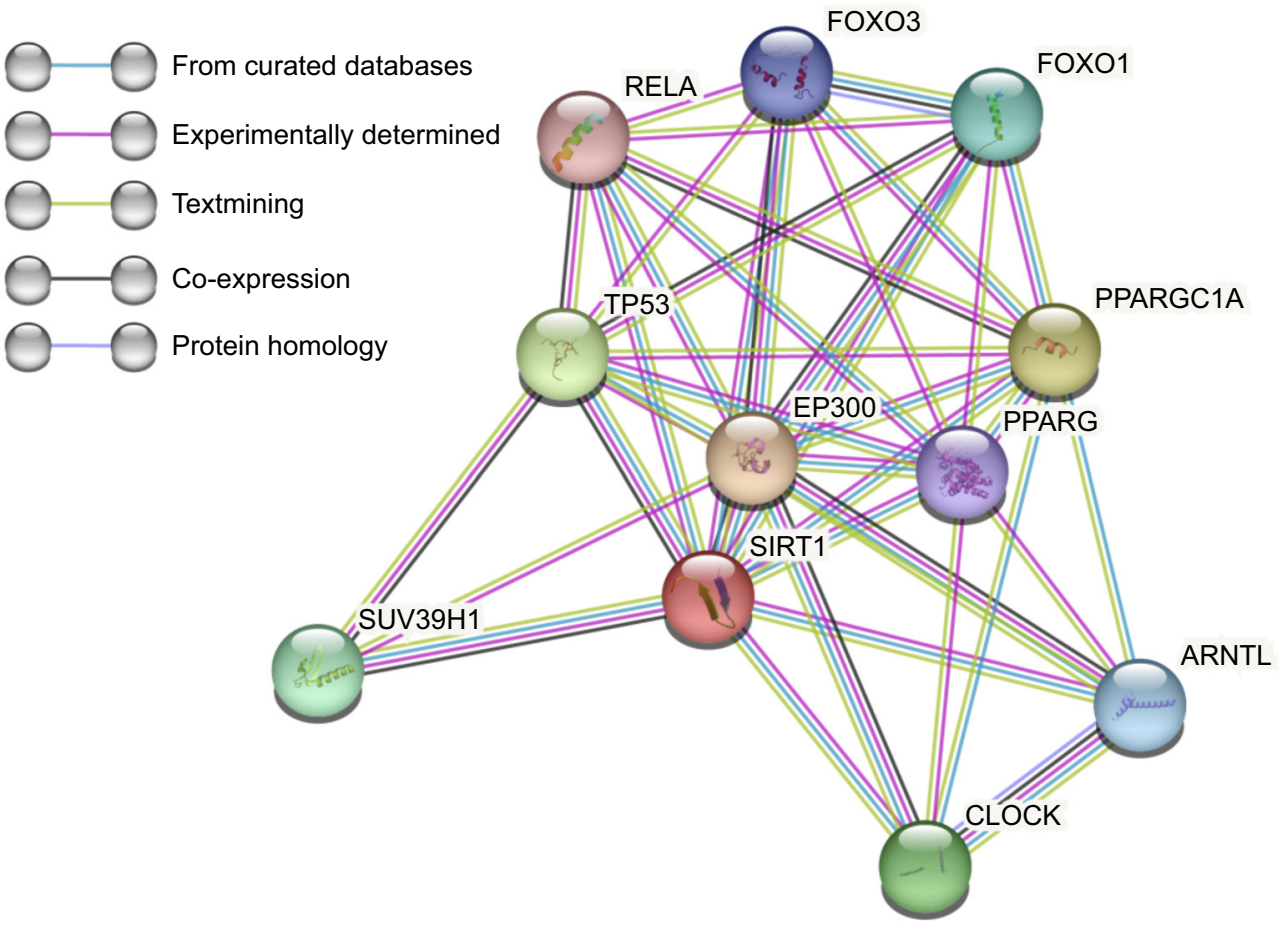
**The protein–protein interaction networks functional enrichment analysis of SIRT1.** Node: Represents individual proteins; Edge: Represents interactions between proteins; Edge color: Indicates the type of interaction; SIRT1: Sirtuin 1.

The excess fatty acids are stored as LDs in the kidney. The accumulation of LD is one of the phenotypes of ccRCC [27]. Sirt1 is associated with lipid metabolism and it is reported that it increases lipophagy and promotes LD catabolism in the liver [28]. The next section of the survey was concerned with the correlation between Sirt1 and lipid metabolism. The Linkedomics database was used to illustrate it [21]. Figure 4 presents the summary statistics for GSEA. There are a series of pathways related to lipid metabolism, such as the TGF-beta signaling pathway, FoxO signaling pathway, and oxidative phosphorylation. At the same time, the protein–protein interaction networks functional enrichment analysis showed part of the functional partners known or predicted to interact with Sirt1 (Figure 5). Among the partners, Peroxisome proliferator-activated receptor gamma coactivator 1-alpha (PPARGC1A) and PPARG control the peroxisomal beta-oxidation pathway of fatty acid and are key regulators of adipocyte differentiation and glucose homeostasis. They have all been proven to be related to Sirt1 (Figure 5). Overall, these results suggest that Sirt1 is highly expressed and regulates the lipid metabolism in ccRCC.

**Figure 6.**
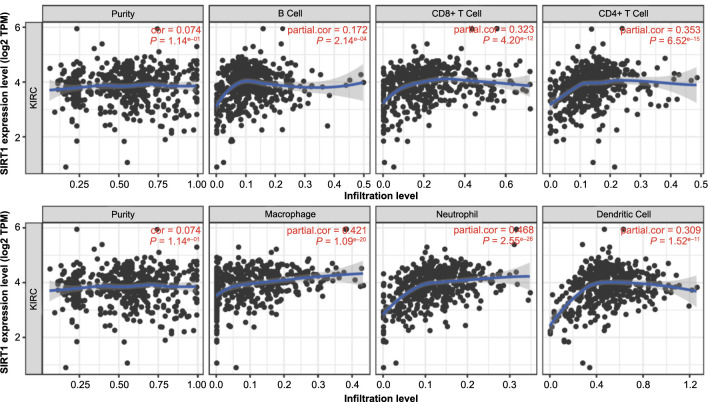
**Sirt1 expression and immune infiltration in ccRCC.** A positive correlation between Sirt1 expression and the presence of B cells, CD8+ T cells, CD4+ T cells, and neutrophils was analyzed by TIMER web. KIRC: Kidney renal clear cell carcinoma; Sirt1: Sirtuin 1; TPM: Transcripts per million; TIMER: Tumor Immune Estimation Resource.

Necrosis is a common occurrence in solid tumor tissue and is often indicative of a poor prognosis [29]. And it is related to inflammation [30]. In order to delve deeper into this relationship, we conducted an analysis of the correlation between Sirt1 expression and immune infiltration in ccRCC using the TIMER website [20]. Our findings indicate a positive correlation between Sirt1 expression and the presence of B cells, CD8+ T cells, CD4+ T cells, and neutrophils (Figure 6). These results suggest that Sirt1 is closely associated with immune infiltration.

**Figure 7.**
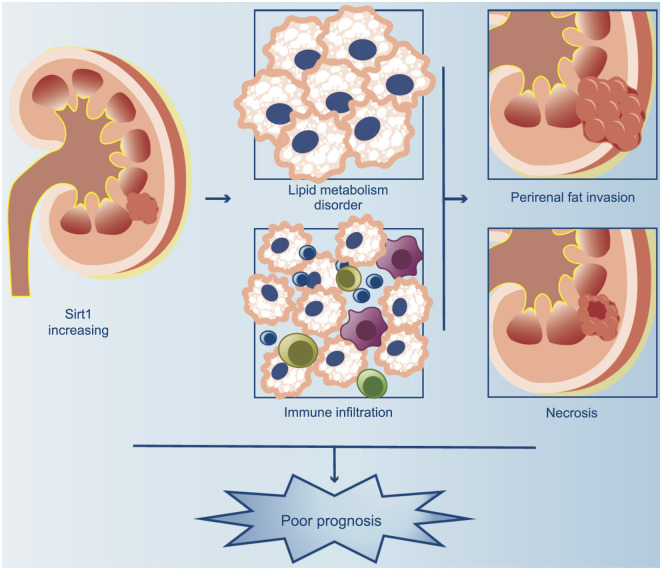
**Effect of Sirt1 expression in ccRCC.** Sirt1 exhibits elevated expression levels in ccRCC. The overexpression of Sirt1 is associated with the occurrence of PFI and necrosis, and linked to disruption of lipid metabolism and necrosis, which lead to an unfavorable prognosis. PFI: Perirenal fat invasion; Sirt1: Sirtuin 1; ccRCC: Clear cell renal cell carcinoma.

In other words, Sirt1 expression disrupts lipid metabolism and immune infiltration and poses a threat to patient survival (Figure 7).

## Discussion

The mortality rate of ccRCC patients remains high even after surgical intervention. The accumulation of lipid dioptids has been linked to carcinogenesis [5], and several studies have explored its underlying mechanisms. For instance, it has been discovered that HIF1α-GPD1 forms a positive feedforward loop that inhibits lipid metabolism in ccRCC [31]. While Sirt1 is a known biomarker of lipid metabolism [4], its role in ccRCC has not been extensively studied. To investigate the role of Sirt1 in ccRCC, we conducted an IHC analysis of Sirt1 expression in 386 ccRCC patients. Almost 80% of cancer tissues expressed Sirt1 but normal tissues did not. Furthermore, patients exhibiting a ccRCC with high Sirt1 expression had significantly worse OS and PFS. It is reported that Sirt1 positively regulates LXRs through deacetylation at lysine K432, and this mechanism appears to play a significant role in cholesterol homeostasis [32]. To further elucidate the function of Sirt1, we attempted to test the fat content in our samples. However, paraffin sections are not suitable for preserving lipids during processing. Therefore, we utilized the Linkedomics database to identify differential genes associated with Sirt1. As shown in Figure 4, the TGF-beta signaling pathway exhibited the most significant positive correlation and has been previously shown to regulate lipid metabolism [33, 34]. Additionally, the FoxO signaling pathway is known to regulate lipid metabolism [35]. Besides, there was a significant negative correlation between Sirt1 and oxidative phosphorylation, which needs fat as part of the fuel for energy [36]. At the same time, the protein–protein interaction networks functional enrichment analysis was built using the Search Tool (Figure 5). PPARGC1A is a coactivator of PPARG, and it plays an important role in lipid metabolism in the kidney [37] and liver [38]. Upregulating the expression level of Sirt1 promoted the deacetylation of PPARGC1A, promoted the transcriptional activity of PPARα, and regulated cholesterol levels [39]. Taken together, these findings suggest a potential association between Sirt1 and lipid metabolism.

In this study, we aimed to investigate the impact of Sirt1 expression on various factors, including age, gender, tumor size, and metastasis. Surprisingly, our results did not reveal any significant differences in Sirt1 expression across these variables. However, we did observe a higher expression of Sirt1 in patients with PFI and necrosis. Numerous studies have attempted to demonstrate that PFI has a poor prognosis [40–42]. And our findings match those of earlier studies. Furthermore, our data certifies that the patients with PFI simultaneously with high Sirt1 expression tend to have a worse prognosis. We hypothesize that Sirt1 may accelerate the fat metabolism, thereby promoting the invasion of PF. To better aid in diagnosis, we constructed clinical models to predict 1-year, 3-year, and 5-year OS and PFS corresponding to patients in different ages, genders, stages, nuclear grades, Sirt1 expression, and presence of PFI and necrosis.

Necrosis is a well-known indicator of poor prognosis in various cancers, including ccRCC [24, 43, 44]. And it is related to inflammation [45]. Previous studies have explored the relationship between Sirt1 and inflammation [46, 47]. And our investigation revealed that Sirt1 expression is significantly correlated with necrosis and immune. Similar to PFI, the combination of necrosis and high Sirt1 expression may serve as a poor prognostic factor in ccRCC. To further elucidate the role of Sirt1 in ccRCC, we analyzed the correlation between Sirt1 expression and immune infiltration levels. Although we did not collect our own data on immune infiltration, we utilized the TIMER web service, which provides reliable data from TCGA [20]. Our findings suggest that Sirt1 expression may enhance immune infiltration in ccRCC. However, further research is necessary to investigate the relationship between Sirt1 expression and immune infiltration in ccRCC.

## Conclusion

Overall, our studies show that Sirt1 is expressed in most ccRCC tissues but not in normal tissues. Increased Sirt1 expression seems linked to PFI, necrosis, immunological infiltration, lipid metabolic problems, and maybe a worse prognosis in ccRCC. Even though our results suggest that Sirt1 may have an impact on PFI, necrosis, and immune infiltration, more research is needed to prove a connection between the elevated Sirt1 expression and these clinical outcomes. Additionally, more research is required to clarify its specific mechanistic role in the development of the disease and the response to treatment. As a result, Sirt1 may be regarded as a prospective therapeutic target for colorectal cancer as well as a possible prognostic marker.

## Data Availability

The authenticity of this article has been validated by uploading the key raw data onto the Research Data Deposit platform (www.researchdata.org.cn), with the approval RDD number as RDDA2024689760.
